# Impact of Plasma Membrane Domains on IgG Fc Receptor Function

**DOI:** 10.3389/fimmu.2020.01320

**Published:** 2020-06-30

**Authors:** Sibel Kara, Lukas Amon, Jennifer J. Lühr, Falk Nimmerjahn, Diana Dudziak, Anja Lux

**Affiliations:** ^1^Department of Biology, Institute of Genetics, Friedrich-Alexander University Erlangen-Nürnberg (FAU), Erlangen, Germany; ^2^Laboratory of Dendritic Cell Biology, Department of Dermatology, University Hospital Erlangen, Friedrich-Alexander University Erlangen-Nürnberg (FAU), Erlangen, Germany; ^3^Division of Nano-Optics, Max-Planck Institute for the Science of Light, Erlangen, Germany; ^4^Medical Immunology Campus Erlangen (MICE), Friedrich-Alexander University Erlangen-Nürnberg (FAU), Erlangen, Germany; ^5^Deutsches Zentrum Immuntherapie (DZI), Erlangen, Germany; ^6^Comprehensive Cancer Center Erlangen-European Metropolitan Area of Nürnberg (CCC ER-EMN), Erlangen, Germany

**Keywords:** type I FcR, type II FcR, FcγR, CLR, cell membrane, lipid rafts, membrane, membrane localization

## Abstract

Lipid cell membranes not only represent the physical boundaries of cells. They also actively participate in many cellular processes. This contribution is facilitated by highly complex mixtures of different lipids and incorporation of various membrane proteins. One group of membrane-associated receptors are Fc receptors (FcRs). These cell-surface receptors are crucial for the activity of most immune cells as they bind immunoglobulins such as immunoglobulin G (IgG). Based on distinct mechanisms of IgG binding, two classes of Fc receptors are now recognized: the canonical type I FcγRs and select C-type lectin receptors newly referred to as type II FcRs. Upon IgG immune complex induced cross-linking, these receptors are known to induce a multitude of cellular effector responses in a cell-type dependent manner, including internalization, antigen processing, and presentation as well as production of cytokines. The response is also determined by specific intracellular signaling domains, allowing FcRs to either positively or negatively modulate immune cell activity. Expression of cell-type specific combinations and numbers of receptors therefore ultimately sets a threshold for induction of effector responses. Mechanistically, receptor cross-linking and localization to lipid rafts, i.e., organized membrane microdomains enriched in intracellular signaling proteins, were proposed as major determinants of initial FcR activation. Given that immune cell membranes might also vary in their lipid compositions, it is reasonable to speculate, that the cell membrane and especially lipid rafts serve as an additional regulator of FcR activity. In this article, we aim to summarize the current knowledge on the interplay of lipid rafts and IgG binding FcRs with a focus on the plasma membrane composition and receptor localization in immune cells, the proposed mechanisms underlying this localization and consequences for FcR function with respect to their immunoregulatory capacity.

## Introduction

Fc receptors (FcR) for immunoglobulin G (IgG) are cell surface receptors widely expressed on cells of both, the innate and the adaptive immune system. Based on distinct mechanisms of IgG binding, these IgG FcRs are currently classified as either type I Fc receptors (classical FcγR) or type II FcRs, which belong to the sugar-binding C-type lectin receptors [reviewed in (1)]. Upon ligand binding, receptors of both families have been shown to induce immune cell activation and cell-type specific effector responses via initiation of distinct intracellular signaling pathways. Ligand-induced cross-linking of multiple receptors facilitating the recruitment of intracellular signaling components was consequently proposed to be a crucial part of receptor function especially for type I Fc receptors [reviewed in (2)]. Since organized membrane microdomains, i.e., lipid rafts, are known to be enriched for signaling components [summarized in (3)], the question arises to what extent IgG FcR function in immune cells is affected by specified membrane domains or the composition of the plasma membrane in general.

## The Cell Membrane is a Highly Complex Lipid Bilayer

**Figure 1:**
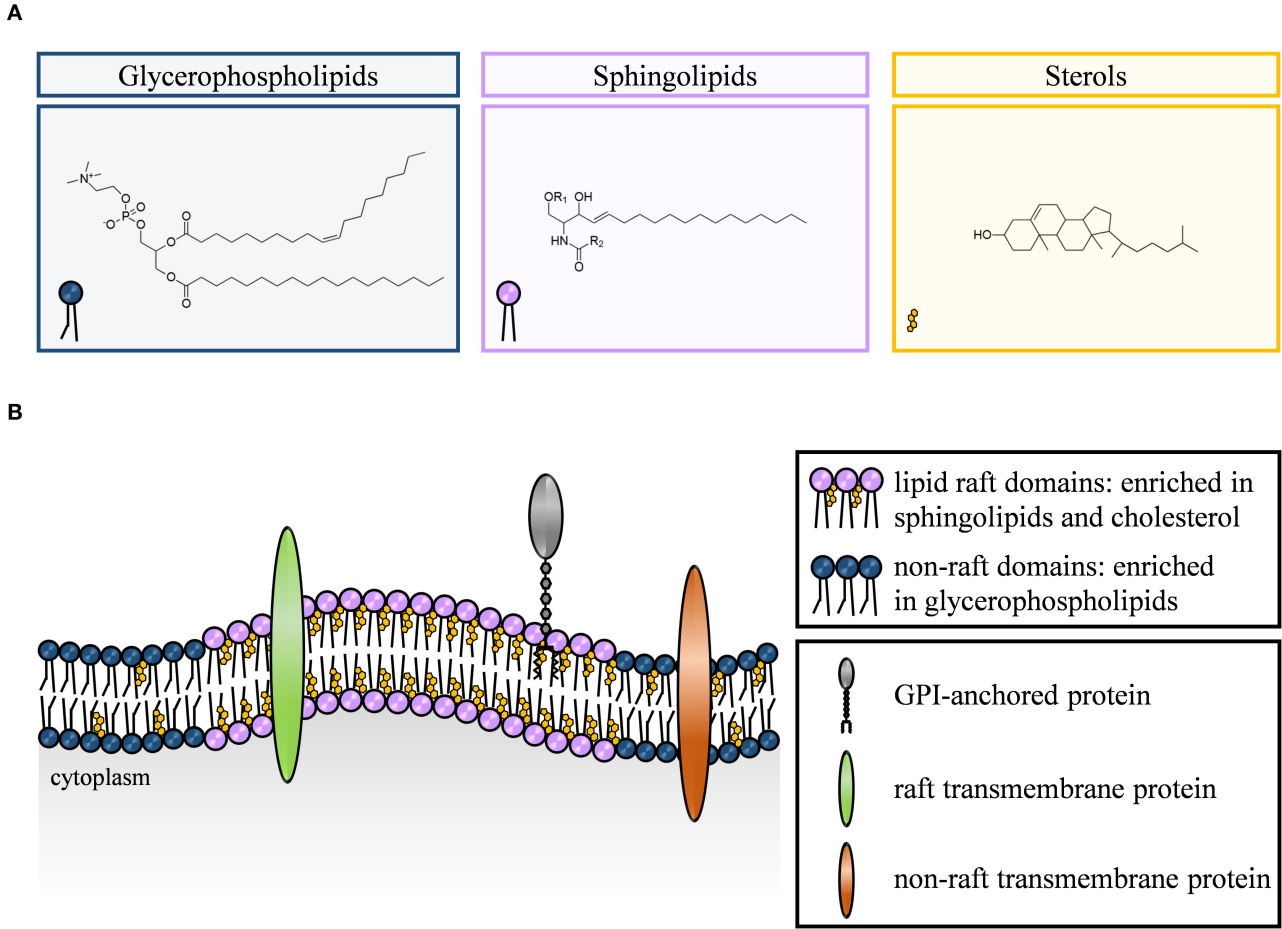
Chemical structure of main lipid classes and composition of cell membranes. The mammalian plasma membrane consists of three lipid classes, namely glycerophospholipids (depicted in blue), sphingolipids (purple), and sterols (yellow) **(A)**. Functionally, the cellular membrane is a lipid bilayer forming both, lipid raft and non-raft domains. Lipid rafts are enriched in sphingolipids, cholesterol, glycosylphosphatidylinositol (GPI)-anchored proteins and some transmembrane proteins, whereas non-raft domains are composed predominantly of glycerophospholipids and non-raft transmembrane proteins **(B)**.

Biomembranes are a prerequisite for cell formation and cell survival as the cellular envelope establishes a biological barrier between intracellular and extracellular space. In more detail, they maintain energy storage, protect the cell from pathogens, are the site for cell-to-cell recognition, and are involved in almost all signaling and membrane trafficking processes by allowing both, active as well as passive transport of molecules into and out of the cell [summarized in (4–7)]. The plasma membrane is likely the most intensely studied biomembrane. In general, it consists of a lipid bilayer composed of a huge variety of different lipids that can be of either amphiphilic or hydrophobic nature, and a multitude of embedded membrane proteins (Figure 1) [for specialized review articles see (4, 8, 9)].

### Structure and Function of Lipid Classes

The lipid composition of the plasma membrane of eukaryotic cells is based on three lipid classes, namely glycerophospholipids, sphingolipids, and sterols.

Glycerophospholipids (e.g., phosphatidylcholine or phosphatidylglycerol) are the predominant lipid component of mammalian plasma membranes and provide their basic framework. Distinct members of this lipid class also operate as second messengers or serve as precursors for the generation of second messengers. A glycerol backbone linked to two hydrophobic fatty acid chains and a phosphate group connected to a hydrophilic alcohol head group are the structural features of glycerophospholipids. Additional structural variations of this lipid species are based on modifications of the head group and the fatty acid chain length, degree of saturation, and linkage to glycerol (4, 10–13).

In comparison to glycerophospholipids, sphingolipids are much less frequent components of lipid cell membranes. However, together with cholesterol, they greatly contribute to the heterogeneity of plasma membrane organization. The common structural feature of sphingolipids is a ceramide core with an amphiphilic sphingosine backbone in turn providing the framework to form more complex sphingolipids such as sphingomyelin or gangliosides (4, 9, 14). Sphingolipids play a critical role both as structural components within membranes as well as bioactive signaling molecules. In this respect, they regulate numerous cellular processes that range from cell growth, differentiation and apoptosis (15, 16) to cytoskeletal reorganization (17, 18).

In recent years, many studies focused on the role of cholesterol in the plasma membrane as a modulator of structural integrity, membrane organization, and fluidity (3, 19, 20). With an amount of up to 40% of all membrane lipids, cholesterol is a major component of mammalian cell membranes (21, 22). It consists of a hydrophilic head group linked to a hydrophobic rigid steroid ring system which serves as a spacer between bulky sphingolipids and promotes the formation of distinct dynamic clusters with higher ordering, i.e., lipid rafts (3).

### From Fluid Mosaic Model to Highly Complex Plasma Membranes

In 1972, Singer and Nicolson postulated the fluid mosaic model of the cell membrane, describing the structure of the plasma membrane as a mosaic of phospholipids, cholesterol, proteins, and carbohydrates. According to this model, the lipid membrane is a neutral two-dimensional solvent, crowded with randomly distributed membrane proteins (23). The fluid mosaic model was based on the observation that most physiological phospholipids have low melting temperatures, suggesting them to preferentially remain in liquid disordered phases. Many studies in the last decades have further refined this model and suggested that mammalian membranes contain very small and dynamic lipid domains with much higher melting temperatures that exist in a liquid ordered phase (24–26) The distribution of liquid ordered and disordered phases subsequently leads to transient formation of membrane nano- or microdomains, so-called lipid rafts that further shape the physical properties of the plasma membrane. Such compartmentalization subsequently facilitates segregation or aggregation of membrane proteins and signaling molecules within these distinct domains thus affecting their biological function (4, 9, 12, 27, 28). It is important to emphasize that both, the lipids in the plasma membrane as well as anchored membrane proteins exhibit a bilateral asymmetric architecture. This asymmetry seems to be required to compensate for membrane perturbations and facilitates membrane reorganization, trafficking and signaling (4, 29, 30). Asymmetry is a consequence of differential distribution of saturated and unsaturated lipids in the plasma membrane bilayer, in which saturated lipids seem to be located exclusively in the exoplasmic leaflet (30, 31). In contrast, heterogeneous cholesterol distribution between the inner and outer leaflet is still debated (21, 22, 32, 33). Membrane bilayer assymetry is further proposed to be modulated by long chain sphingolipids (34).

### Lipid Rafts: Multifunctional Communication Platforms in Health and Disease

Lipid rafts are small, tightly packed and highly organized but dynamic membrane domains enriched in cholesterol and sphingolipids. The lipid raft hypothesis proposes that organized domains are fluid at physiological temperatures, facilitating lateral diffusion of proteins and lipids within the domain as well as of the raft itself. Furthermore, select interactions between cholesterol, saturated, and glycosylated lipids are involved in the recruitment of other lipids and proteins, e.g., glycosylphosphatidylinositol (GPI)-anchored and acylated or palmitoylated transmembrane proteins (9, 35–39).

From a functional perspective, lipid rafts are debated to be involved in sorting of membrane proteins, protein trafficking, membrane partitioning, and signaling (28, 40–43). In this respect, immunological synapses formed as interfaces between activated lymphocytes and their cognate antigen presenting cells have been hypothesized to be an example of organized raft domains within the immune system (44–46). Furthermore, lipid rafts are also discussed to play a crucial role in hematopoietic stem cell homing (47–49), mobilization (47, 50, 51), and differentiation (52–54). Moreover, many studies indicate lipid rafts to be involved in various diseases, e.g., HIV-1 infection including a role in virus budding (55, 56), cancer (57), or neurodegenerative disorders such as prion diseases (58), and Alzheimer's (59, 60).

### Lipid Composition of Immune Cells

In the last years, evolutionary details have been gathered about the lipidome of mammalian tissues, e.g., cortex, brain, heart, kidney, muscle, or liver (61, 62) and mammary breast cancer cells (63, 64). But despite the fact that the lipid membrane plays a crucial role in many processes involved in immune cell function, information on the composition of immune cell membranes is surprisingly scarce. Recently, novel approaches allow for the detailed analysis of plasma membrane lipids by lipidomics, a method based on sensitive high-throughput mass spectrometry (65–67). Lipidomics analysis revealed, for example, that the membrane lipid composition of leukocytes is characterized by sizable amounts of cholesterol and phosphatidylcholine. However, the observed amounts were lower than in neurons or epithelial cells (38). A study by Leidl et al. provided more detailed information on the lipidome of primary human peripheral blood leukocytes including neutrophils, monocytes, and lymphocytes. Accordingly, elevated amounts of ceramide and cholesterol but diminished phosphatidylcholine and sphingomyelin levels were identified in human neutrophils in comparison to monocytes. Lymphocytes exhibited an even lower content of phosphatidylcholine and sphingomyelin in contrast to monocytes and reduced levels of ceramide and cholesterol as compared to neutrophils. Thus, the authors propose monocytes and to a lesser degree lymphocytes to possess more fluid and neutrophils more rigid membrane features (65). In addition, the lipidome of human monocyte-derived DCs (moDCs) has been analyzed in both resting cells and in cells stimulated with the pro-inflammatory cytokine interleukin 17A (IL-17A). IL-17A appears to remodel the lipid metabolism by increasing the phospholipid and cholesterol content in moDCs and facilitates the formation of foamy rather than resting cells. Consequently, Salvatore et al. suggest that IL-17A activated lipid-rich moDCs might be involved in atherosclerosis (68).

Differences in the lipid membrane composition of immune cells have also been observed with respect to the sialic acid containing gangliosides [summarized in (69)]. For example, GM3 is the sole ganglioside found in the membrane of hematopoietic stem cells (70), dendritic cells [summarized in (71)], macrophages (72, 73), and monocytes (72, 74). In contrast, mast cells (75, 76), B cells (77, 78), and T cells (78–80) possess GM1, GM3 as well as GD3 in their cell membranes. Furthermore, neutrophils contain GM1 and GM3, but no GD3 (81–83), whereas NK cells display GM1 and GD3, but lack GM3 (79, 80).

Although these kinds of analysis provide detailed information about the individual lipid species contributing to membrane formation, it is difficult to predict physical properties of membranes from the lipid composition alone. Nevertheless, it became increasingly clear that specific properties of immune cells such as cell rolling and adhesion to endothelial cells are indeed affected by the lipid cell membrane composition (84–87).

Membrane composition and lipid rafts have also been shown to impact many different families of immune receptors including toll-like receptors (TLR) (88, 89), B cell receptors (90–92) and T cell receptors (93, 94), while negatively regulating elements such as transmembrane phosphatases by exclusion from lipid rafts (9). It is however much less clear to what extent lipid cell membranes influence the immunoregulatory function of the diverse family of IgG binding FcRs, both with respect to ligand binding as well as intracellular signal transduction.

## Characteristics, Expression and Signaling of Type I FC Receptors

Classical FcγRs, or type I Fc receptors, interact with the fragment crystallizable (Fc) of IgG and thereby provide the crucial link between the soluble effector molecules of an adaptive immune response and innate immune effector cells. In a cell-type dependent manner, FcγR can trigger a multitude of different IgG effector functions including the uptake of IgG-coated pathogens by phagocytic cells, enhancement of antigen-presentation by dendritic cells (DCs), innate immune cell activation, and production of inflammatory mediators or killing of IgG coated target cells by antibody-dependent cytotoxicity (ADCC). In turn, FcγR expression on B cells is involved in modulation of adaptive immune responses and induction of apoptosis [summarized in (2)]. In humans, these various functions are maintained by a family of different FcγRs (FcγRIa/CD64A, FcγRIIa/CD32A, FcγRIIb/CD32B, FcγRIIIa/CD16A, and FcγRIIIb/CD16B) whose members are characterized by distinct expression profiles, IgG binding capacity and intracellular signaling potentials.

### Expression Patterns of Type I Fc Receptors

One major layer of complexity in FcγR biology is cell-type specific expression of different FcγRs. As summarized in Table 1, typical examples of the latter include neutrophils expressing FcγRIIa and FcγRIIIb (and FcγRI upon activation), classical monocytes expressing FcγRIa, FcγRIIa, and FcγRIIb, and non-classical monocytes expressing FcγRIIa, FcγRIIb, and FcγRIIIa. FcγRIIa is also found on eosinophils that may express FcγRI upon stimulation, basophils that strongly co-express FcγRIIb and mast cells co-expressing FcγRIIb and FcγRIIIa (95, 96). Human DCs have been shown to express predominantly FcγRIIa/b and FcγRIa (97, 98) in a presumably DC subtype specific manner while expression of FcγRIIIa is still under discussion (99, 100). In contrast, NK cells exclusively express FcγRIIIa while FcγRIIb is the only FcγR found on B cells (95). While T cells largely lack FcγR expression there is accumulating data suggesting that select T helper cell subsets may express the inhibitory FcγRIIb [reviewed in (95, 101)].

**Table 1:** Expression pattern of type I and type II Fc receptors on human immune cell populations (*induced upon cell activation, ^‡^ monocyte subpopulations not distinguished, n.d., not determined).

		**B cells**	**T cells**	**Classical monocytes**	**Non − classical monocytes**	**NK cells**	**Neutrophils**	**Eosinophils**	**Basophils**	**Mast cells**	**Macrophages**	**Conventional DCs**	**Plasmacytoid DCs**
Type I	FcγRIa	–	–	+	–	–	+*	+*	–	+*	+	+	–
	FcγRIIa	–	–	+	+	–	+	+	+	+	+	+	+
	FcγRIIb	+	–	+	+	–	+	–	+	+	+	+	+
	FcγRIIIa	–	–	–	+	+	–	–	–	+	+	–	–
	FcγRIIIb	–	–	–	–	–	+	–	–	–	–	–	–
Type II	Dectin-1	+	+	+	+	–	+	+	n.d.	+	+	+	+
	DC-SIGN	–	–	–	–	n.d.	–	–	–	n.d.	+	+/–	–
	FcεRII	+	+*	+^‡^	+^‡^	n.d.	+*	+*	n.d.	n.d.	+*	n.d.	n.d.

### Structure of FcγRs

**Figure 2:**
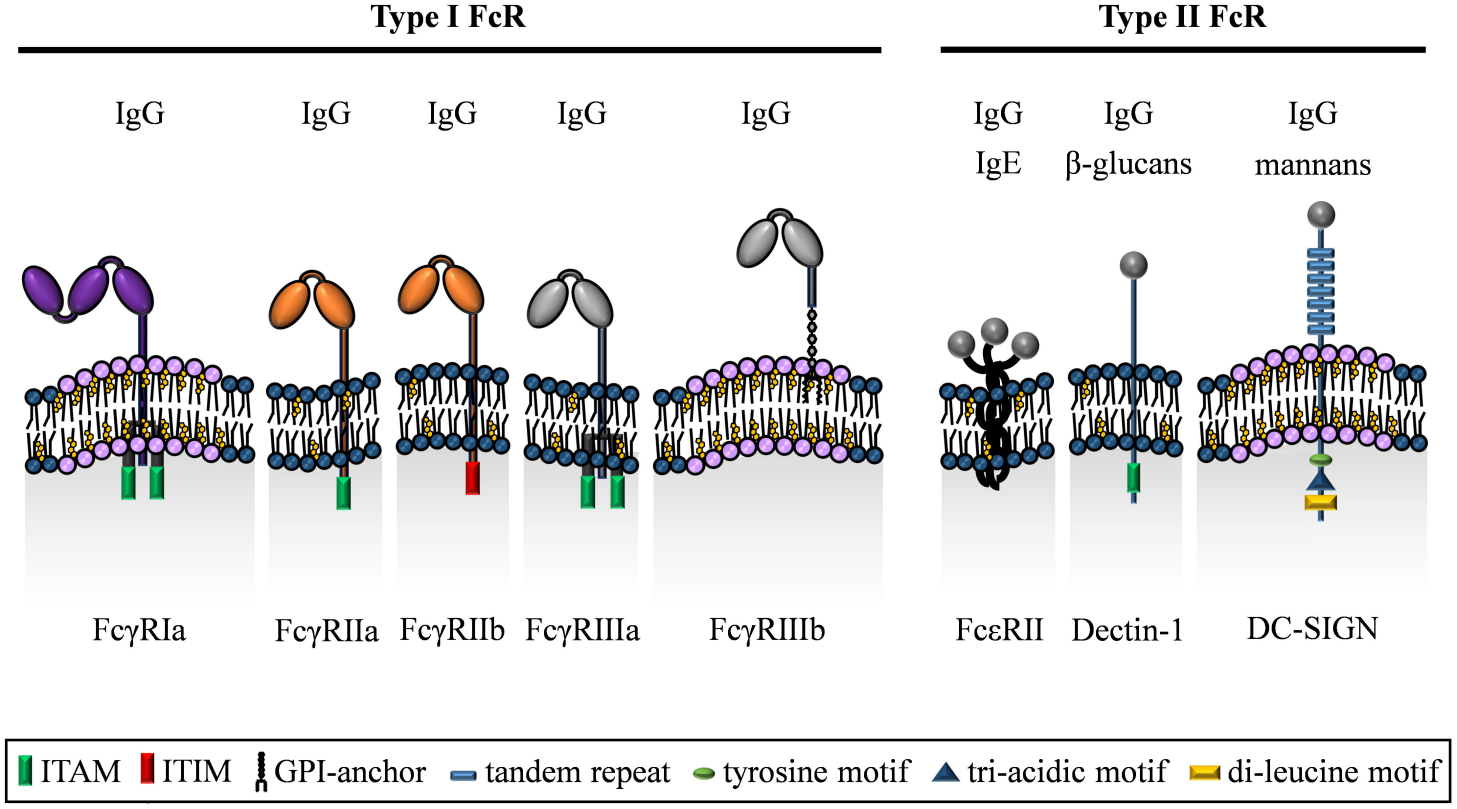
The family of IgG binding type I and type II Fc receptors. The Fc receptors for IgG can be distinguished by their distinct binding mechanisms into either type I FcRs (classical FcγR) or type II FcRs (C-type lectin receptors). With respect to signal transduction, FcγRIa, FcγRIIa, FcγRIIIa, and Dectin-1 signal via ITAM domains in their cytoplasmic tail or on the associated FcεRγ chain. In contrast, the only inhibitory receptor FcγRIIb carries an ITIM domain in its cytoplasmic region. In addition, the GPI-linked FcγRIIIb lacks an intracellular signaling domain, but has a crucial role in binding of IgG immune complexes and enhances signaling upon cross-linking with other FcγRs. Besides, FcεRII has a short cytoplasmic region, whereas DC-SIGN exhibits a cytoplasmic domain including tyrosine, di-leucine and tri-acidic motifs. In addition to recognizing IgG, Dectin-1 and DC-SIGN are known to bind other ligands including defined sugar moieties.

The human FcγR family is predominantly composed of type I transmembrane proteins (Figure 2). The only known exception is FcγRIIIb that is anchored to the cell membrane via a glycosylphosphatidyl-inositol (GPI) tail (102). Otherwise, the FcγR α-chain is composed of an aminoterminal extracellular domain involved in binding IgG, a transmembrane-spanning domain and a carboxyterminal intracellular domain. With the exception of FcγRIIa, activating FcγRs require the association with the FcεRγ chain (short FcRγ) for cell surface expression (103–105), a signaling competent accessory chain originally discovered for its role in expression and function of the IgE FcR (FcεR) (106). The FcγR-FcεRγ chain complex assembles by non-covalent interactions of the respective transmembrane domains and is indispensable for cell surface expression as well as the immunomodulatory FcγR functions (107).

### Classification of FcγRs According to Affinity for IgG or Intracellular Signaling

Based on their affinity for IgG, the high-affinity FcγRIa can be distinguished from the low-to-medium affinity FcγRII and FcγRIII family members. Consequently, FcγRIa is able to interact with monomeric IgG (108), whereas FcγRIIa/b and FcγRIIIa/b require multiple IgG molecules in complex, i.e., IgG immune complexes (IgG-IC) typically obtained by IgG recognizing their cognate antigens. The resulting multivalency of the interaction is able to compensate for a reduced affinity of an individual interaction (109, 110).

Alternatively, human FcγRs can be grouped by their intracellular signaling potential, i.e., what type of intracellular signaling pathway they are able to initiate. Due to the presence of an intracellular immunoreceptor tyrosine activation motif (ITAM), three activating FcγRs are recognized (FcγRIa, FcγRIIa, and FcγRIIIa). Within these receptors, the ITAM is either encoded by the cytoplasmic domain of the ligand-binding FcγR α-chain (as is the case for FcγRIIa) or is part of the accessory FcεRγ chain (FcγRIa and FcγRIIIa). In contrast, the FcγRIIb α-chain contains an immunoreceptor tyrosine based inhibitory motif (ITIM) rendering it an inhibitory receptor [reviewed in (2)]. The GPI-linked FcγRIIIb, which is only found in humans, is a signaling-deficient FcγR since it neither contains an intracellular domain nor interacts with FcεRγ. However, it plays a major role for capture of IgG immune complexes and co-operates with FcγRIIa for induction of signaling (111–113).

### Intracellular Signaling of FcγRs

Albeit FcγR pose a quite diverse receptor family with respect to expression and structural composition, their intracellular signaling pathways are surprisingly similar. Upon IgG recognition, the intracellular ITAMs of activating FcγRs are phosphorylated by kinases of the SRC family enabling recruitment of SYK kinases. Downstream signaling includes activation of phosphoinositide 3-kinase (PI3K), which causes the release of intracellular calcium via activation of phospholipase Cγ (PLCγ). In addition, FcγRs trigger the mitogen-activated protein kinase (MAPK) pathway via ras and raf. Since most immune cells co-express both, activating and inhibitory FcγRs, IgG-IC binding will also initiate intracellular inhibitory signaling via FcγRIIB. Upon phosphorylation of ITIMs, phosphatases, e.g., SRC-homology-2-domain-containing inositol-5-phosphatase (SHIP) are recruited that interfere with the activating pathways by reducing the activation of ras and by hydrolysis of phosphoinositide intermediates [summarized in (114)]. Accordingly, activating and inhibitory FcγRs cooperate in setting a threshold for immune cell activation.

## Properties and Expression of Type II FC Receptors

Type I and type II Fc receptors share the ability to bind to IgG Fc but their mode of interaction differs. Based on the composition of the coupled bi-antennary Fc glycan, type I Fc receptors recognize IgG Fc in an open protein conformation at equimolar rate. In contrast, closed conformation recognition via type II receptors enables the binding of two Fc fragments at the same time (1, 115). So far, the identified type II Fc receptors belong to the super family of C-type lectins receptors (CLRs).

### Classification of C-Type Lectin Receptors

The classification of CLRs was first established to separate Ca^2+^-dependent (C-type) from independent lectins [reviewed in (116)] where all Ca^2+^-dependent lectins share an intrinsic carbohydrate recognition domain (CRD) allowing for binding of foreign and endogenous carbohydrate ligands (117). Owing to conserved residue motifs and the unique folding of the CRD, it was termed C-type lectin domain, which is the defining hallmark feature of all CLR family members (116). However, sequence analysis identified new CLRs able to recognize other ligands then carbohydrates including exogenous and endogenous protein side chains, glycosphingolipids and inorganic ligands in an Ca^2+^-dependent or -independent manner [for specialized review articles see (118–120)]. Therefore, not all CLRs are classical pattern recognition receptors. Today, the CLR super family comprises more than one thousand soluble or membrane bound members (119).

Besides the C-type lectin domain, membrane bound CLRs are further dissected into type I and type II CLRs (not to be confused with type I and type II FcRs) depending on the localization of the N-terminus. They further differ in their intracellular motifs responsible for receptor internalization, cycling and signaling [summarized in (121)]. Because of this complexity, here we will focus only on the three so far described type II FcRs, DC-SIGN (CD209), Dectin-1 (CLEC7A) and Fcε receptor II (FcεRII; CD23) characterized by the ability to bind immunoglobulins (Figure 2) although this is a current matter of debate (119, 122–125).

### Dendritic-Cell-Specific ICAM-3 Grabbing Non-integrin (DC-SIGN)

DC-SIGN is a type II CLR carrying an extracellular, C-terminal CRD, followed by seven complete and one incomplete tandem repeats and a hydrophobic, intracellular N-terminus. The latter is including tyrosine-, di-leucine and tri-acidic motifs, which are involved in internalization and shuttling (120, 121, 126, 127). DC-SIGN is specifically expressed on moDCs and macrophage subpopulations including alveolar macrophages (128, 129) (Table 1). In addition, expression of DC-SIGN on conventional DCs is also proposed (130). Following ligand recognition, it was demonstrated that DC-SIGN is rapidly internalized to late endosomes and lysosomes (127). The highly conserved Glu-Pro-Asn CRD motif facilitates the recognition of structures containing several mannose- and fucose-residue bearing ligands from pathogens, allergens, or endogenous molecules (120). One of the most prone antigens recognized by DC-SIGN is the HIV glycoprotein gp120 (126, 131, 132).

Signaling mediated via DC-SIGN is independent of immunoreceptor tyrosine-based motifs and does not lead to major modulations of immune cell activation by itself. DC-SIGN rather co-operates and fine-tunes the signaling of other receptors, e.g., TLRs (120). In this respect, high mannose ligands were found to foster the assembly of LSP1, KSR1, and CNK ultimately boosting the transcription of CXCL8, IL-12, IL-6, and IL-10 promoters via ras, raf-1 (133) and the NF-κB p65 subunit (133, 134). In contrast, fucose-rich ligands only allowed for DC-SIGN association with LSP1 leading to production of IL-10 in a raf-1 independent manner and negatively influenced transcription of IL-6 and IL-12 (133). Finally, the DC-SIGN agonist Salp15 was found to negatively regulate the production of TNFα, IL-6 and IL-12 via ras and raf-1 dependent activation of MEK (135).

Besides the carbohydrate binding capacity, DC-SIGN has been originally described to be important for the emigration of moDCs from vascular endothelium (128). Moreover, DC-SIGN is a key player in establishing contacts between DCs and resting T cells (136). Besides this multitude of functions, DC-SIGN was identified as the first receptor being able to bind sialylated IgG. Sialylated IgG Fc, which is a component of intravenous immunoglobulin G (IVIg) preparations (137), was demonstrated to bind to DC-SIGN on macrophages and thereby mediate anti-inflammatory responses *in vivo* (138, 139). Thus, sialylated IgG Fc may comprise the therapeutically active component in IVIg preparations even though the exact mechanisms are still subject of debate (124, 137, 140, 141).

### Dendritic-Cell-Associated C-Type Lectin 1 (Dectin-1)

Dectin-1 serves as a prototypic model for CLR pattern recognition receptors as its functions range from pathogen recognition to the initiation of signaling and uptake of bound material. Thus, Dectin-1 facilitates the recognition of β-1,3- and β-1,6-glucans in the defense against fungi in a Ca^2+^-independent manner (142–144). It was recently also shown to recognize annexins on apoptotic cells in mice, thereby contributing to self-tolerance (145) albeit it remains to be determined if this finding can be translated to the human system. Karsten et al. showed that IgG1 immune complexes counteract C5a receptor signaling via FcγRIIb and Dectin-1 (146). Even though this study does not demonstrate direct IgG binding to Dectin-1, it has subsequently been suggested that Dectin-1 may recognize the core-fucose residue of the IgG sugar moiety (123).

As summarized in Table 1, Dectin-1 is widely expressed on human innate cells including both monocyte populations, conventional and plasmacytoid DCs, macrophages, neutrophils, eosinophils and mast cells. In addition, B cells and a subpopulation of T cells have also been suggested to express Dectin-1 (142, 143, 147, 148).

Full length Dectin-1 is composed of an extracellular C-terminus carrying a single CRD followed by a stalk region, the transmembrane domain and an intracellular N-terminus carrying an ITAM motif (149, 150). Smaller spliced isoforms of Dectin-1 lacking, e.g., the stalk region are expressed in a cell-type specific manner (142, 149, 151, 152).

Following ligand recognition, Dectin-1 has been shown to recruit syk via its hemITAM (single tyrosine based ITAM) motif initiating the production of reactive oxygen species, act in the activation of the NLRP3 inflammasome and the canonical p65 NF-κB pathway (120, 153–155). Comparable to DC-SIGN, Dectin-1 was also found to foster the syk-independent Raf-1 pathway (133). Finally, Dectin-1 signaling can synergize with MyD88 dependent TLR signaling in the induction of NF-κB (150).

### The Low Affinity IgE Receptor FcεRII (CD23)

FcεRII (or CD23) is a type II CLR carrying an extracellular C-terminal CRD, a stalk region important for CD23 oligomerization, another extracellular region of yet unknown function, one single hydrophobic membrane domain and a short cytoplasmic N-terminus (156, 157). Thus, FcεRII is the only Fc receptor not belonging to the immunoglobulin superfamily (156). Described ligands for FcεRII are IgE, CD21, CD11b, CD11c, but also IgG (1, 158, 159). FcεRII is thought to be expressed on B cells, T cells, polymorphonuclear leukocytes including eosinophils and neutrophils, monocytes, follicular DCs as well as epithelial and stromal cells (156, 160–165) (Table 1).

Structure and function of FcεRII strongly differ from the high affinity IgE receptor FcεRI as it consists of a single amino acid chain, is not associated with the FcεRγ chain and is found in trimers on the cell surface (164, 166, 167). Ligation of FcεRII on B cells downregulates IgE production in the latter (156). FcεRII can also be released from the cell surface by metalloproteinases to exert cytokine-like activities while maintaining its IgE binding activity (168). These soluble FcεRII:IgE complexes can then interact with surface bound IgE receptors, thereby positively acting on survival and differentiation of B cells (169–171). Furthermore, soluble FcεRII was found to ligate CD11b and CD11c thereby promoting NF-κB dependent inflammatory responses by human monocytes including nitric oxide production, cAMP synthesis and cytokine production (172).

Depending on external environmental stimuli, FcεRII exists in two isoforms differing in their amino-terminal sequence and transcriptional start sites (156). While FcεRIIa is constitutively expressed on B cells, FcεRIIb expression may be induced and foster antigen retention following binding instead of processing and endocytosis (156). Membrane-bound FcεRII was further described to be involved in B cell selection and affinity maturation (173). This is mediated by autocrine upregulation of FcγRIIb following binding of sialylated Fc domains to FcεRII, thereby increasing the threshold for BCR signaling (173).

In summary, DC-SIGN, Dectin-1, and FcεRII are considered to be type II Fc receptors due to their capacity to interact with immunoglobulins. All of them belong to the superfamily of CLRs unified by the structural feature of the C-type lectin domain. Nevertheless, it has at least to be noted that the IgG binding capability of DC-SIGN and FcεRII has recently been challenged (125).

## Type I FC Receptors Differentially Localize Within the Cell Membrane

Circulating human immune cells exhibit different FcγR expression patterns, enabling the cells to implement cell-type specific regulatory functions [reviewed in (2)]. In recent years, many studies focused on the role of lipid rafts in modulation of FcγR activity.

### FcγRIa

Several studies indicate that FcγRIa is predominantly located within lipid rafts (174–176). In myeloid cell lines, high amounts of FcγRIa are detected in detergent-resistant membrane fractions. Moreover, imaging studies of primary human monocytes show co-localization of FcγRIa with GM1 (monosialotetrahexosylganglioside) ganglioside, a marker (177) that predominantly partitions to lipid rafts. According to a study by Beekman et al., this localization is proposed to be independent of FcγRIa cross-linking (174). In accordance, a more detailed imaging study focusing on the mechanism of FcγRIa membrane localization, identifies patching of FcγRIa and the membrane-associated protein 4.1G (178, 179) in absence of ligand binding (175). Cross-linking of FcγRIa induces cytoplasmic serine phosphorylation and dissociation from 4.1G enabling FcγRIa to remain in lipid rafts. The authors propose that 4.1G plays a crucial role in targeting FcγRIa to lipid rafts in a serine phosphorylation dependent manner (175). Association of FcγRIa with lipid rafts is indeed important for FcγRIa function, e.g., in contributing to antimicrobial activity (176) or involvement in the secretion of TNF family cytokines (175). Cholesterol depletion is a common experimental method to disrupt lipid raft organization [reviewed in (40, 180)] and has been shown to alter FcγRIa arrangement in the cell membrane (174). With respect to IgG binding, cholesterol depletion results in a diminished capability of FcγRIa to recognize monomeric IgG, while interaction with IgG immune complexes is unaltered. Cross-linking induced signal transduction seems therefore not to be affected by FcγRIa relocalization to lipid rafts (174).

### FcγRIIa

**Figure 3:**
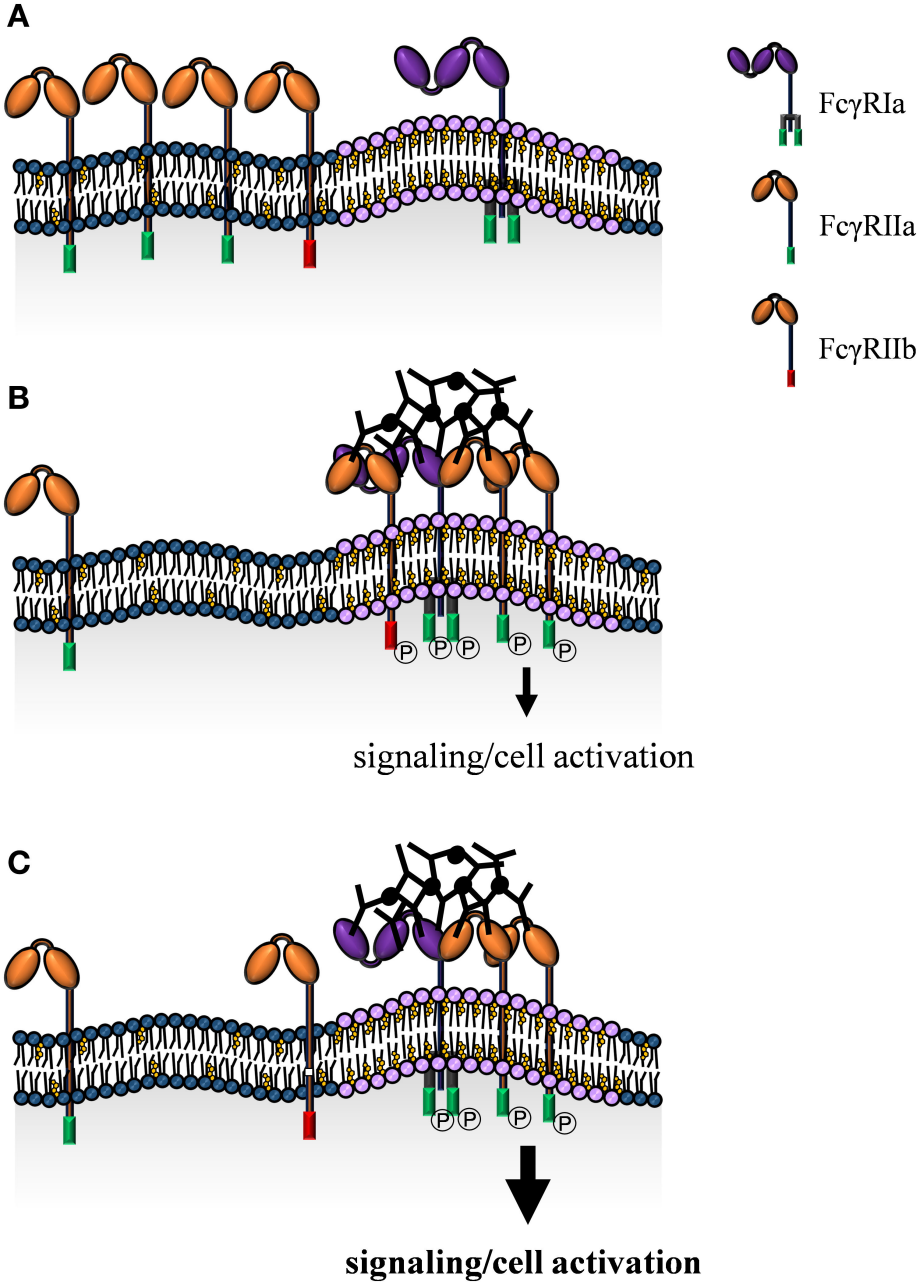
Model of FcγR localization and receptor clustering in the cell membrane. Classical monocytes express the activating FcγRIa and FcγRIIa and the inhibitory FcγRIIb with distinct membrane localizations. FcγRIa is predominantly confined to cholesterol-rich lipid raft domains, while FcγRIIa and FcγRIIb are found in non-raft regions in absence of IgG immune complexes **(A)**. Upon IgG immune complex binding FcγRIIa/b are recruited to lipid rafts. Close proximity to each other and to signaling components concentrated at lipid raft domains, enables ITAM/ITIM phosphorylation and induction of intracellular signaling cascades **(B)**. Exclusion of FcγRIIb from lipid rafts due to a mutation in the transmembrane domain causes enhanced activatory signaling **(C)**.

Activating FcγRIIa is the most studied FcγR with respect to membrane localization and its underlying mechanisms. However, consistency of the data is complicated by the use of different experimental model systems. FcγRIIa is widely expressed on innate immune cells (including neutrophils and both monocyte subsets). These cell types may also express the closely related inhibitory FcγRIIb (95, 96). This further complicates interpretation of data because of high homology of the extracellular domains. Consequently, many available monoclonal antibodies are not able to discriminate between FcγRIIa and FcγRIIb (181). Regardless of these open questions it was suggested, that FcγRIIa is dispersed in the cell membrane and is localized to lipid raft domains upon IgG-mediated cross-linking (182–184) (Figure 3). Of note, lipid raft localization is apparently not simply a consequence of FcγRIIa cross-linking. In fact, interaction with IgG correlates with FcγRIIa localization as both, lipid raft disruption as well as receptor mutants with abolished lipid raft re-localization show decreased IgG binding (185–187) and IgG immune complex phagocytosis (186). However, data regarding phagocytosis is controversial, since García-García et al. show a more pronounced uptake in absence of lipid raft localization (188).

Upon receptor cross-linking, intracellular signaling is triggered by phosphorylation of ITAMs and recruitment of intracellular signaling molecules. Lipid raft localization indeed seems to enhance FcγRIIa phosphorylation (184, 189, 190) and increasing sphingolipid-cholesterol-rich membrane rafts causes FcγRIIa clustering and phosphorylation even in absence of IgG (191). Furthermore, cholesterol depletion is consistent with abolished association of cross-linked FcγRIIa with low density detergent resistant membranes and abrogates FcγRIIa tyrosine phosphorylation (189). In contrast, Barabe et al. observed prominent FcγRIIa localization in non-raft domains in human primary neutrophils even upon cross-linking-induced clustering of FcγIIa. According to this study, only a small portion of FcγRIIa rearranges in rafts, where it is degraded in a short period of time. Interestingly, this can be prevented by cholesterol depletion. Nevertheless, this neither results in diminished tyrosine phosphorylation nor calcium release, indicating that the integrity of cholesterol-rich raft domains and therefore translocation of FcγRIIa in lipid rafts seems not to be critical for FcγRIIa-mediated signal transduction (192) or cellular effector functions such as superoxide generation (183). This conclusion is in part supported by García-García et al. showing FcγRIIa-induced syk activation independent of lipid rafts. Of note, the same authors also propose NF-κB activation to be dependent on lipid rafts (188).

Mechanistically, some studies propose palmitoylation of a cytoplasmic cysteine residue to be responsible for FcγRIIa membrane localization to ordered domains (185, 187, 193). However, this was not confirmed by García-García et al., who instead suggest the transmembrane domain to be crucial for receptor localization (188). In any case, phosphorylation of the FcγRIIa ITAM seems not to be involved mechanistically in lipid raft recruitment (183, 194).

### FcγRIIb

The inhibitory receptor FcγRIIb is mainly expressed on B cells and monocytes and plays a crucial role in modulating activating immune receptors and thereby immune cell activation essentially preventing autoimmune diseases. There is convincing data that lipid raft localization of FcγRIIb is initiated by ligand binding (195) and seems to be critical for receptor function. In fact, FcγRIIb with a specific mutation in the transmembrane domain (Ile232Thr) is not able to localize to lipid rafts and is associated with an increased susceptibility for autoimmune diseases due to attenuated negative regulation of immune cells (196, 197) (Figure 3). Mechanistically, cross-linking of the B cell receptor and FcγRIIb enhances FcγRIIb recruitment to lipid rafts (195), where B cell receptor signaling induces transphosphorylation of FcγRIIb (196). The extent of this tyrosine phosphorylation is a decisive indicator for distribution of the receptor within organized domains. Accordingly, the magnitude of inhibition of B cell receptor signaling corresponds to the level of FcγRIIb recruitment within lipid rafts (196). These findings are further supported by imaging studies of living cells using confocal as well as total internal reflection fluorescence (TIRF) microscopy in combination with fluorescence resonance energy transfer (FRET) that not only show co-localization of the B cell receptor and FcγRIIb in lipid rafts, but also destabilization of the complex and inhibition of immune synapse formation upon receptor cross-linking (198).

### FcγRIIIa

Albeit FcγRIIIa is found to be expressed on NK cells and a small monocyte subpopulation, so-called non-classical monocytes, the membrane localization of FcγRIIIa has only been studied in NK cells. For this cell type, cellular fractionation as well as confocal microscopy revealed that FcγRIIIa preferentially resides in non-raft membrane regions in resting cells compared to enhanced localization in lipid rafts upon cell stimulation (199, 200). Cross-linking with IgG leads to recruitment of FcγRIIIa to lipid rafts, induction of intracellular signaling (200) and actin-dependent internalization of FcγRIIIa (201). Further supporting the importance of lipid raft association, FcγRIIIa signaling is inhibited in response to chemical disruption of organized domains even upon co-stimulation of NK cells (200). Mechanistically, FcγRIIIa redistribution to organized domains seems to be dependent on PLC activity (201). Lou et al. even suggested the presence of a positive feedback loop of receptor cross-linking, raft aggregation and signal transduction. In turn, killer cell immunoglobulin-like receptors (KIR) negatively affect formation and rearrangement of organized domains through (SHP-1 induced) dephosphorylation of signaling molecules and diminished cell polarization (202). Therefore, it was suggested that polarization and consequently FcγRIIIa relocalization to lipid rafts is indeed required to initialize FcγRIIIa signaling (200).

### FcγRIIIb

Lipid-lipid interactions in highly organized raft domains contribute to the recruitment of GPI-anchored proteins (25). The only member of the FcγR family that is inserted into lipid rafts by a GPI linker is FcγRIIIb, which is exclusively expressed on the cell surface of neutrophils (203). Membrane fractionation experiments determined that FcγRIIIb indeed partitions in high density sphingolipid and cholesterol-rich membrane domains (204). Strikingly, signal transduction can be initialized by clustering of the GPI-anchored FcγRIIIb itself in absence of any characteristic signaling motifs (26) and is even enhanced upon cross-linking of FcγRIIIb with other receptors, e.g., FcγRIIa (205). The function of membrane anchored FcγRIIIb in signal transduction is however ambiguous. Besides activating intracellular signaling, FcγRIIIb is strikingly able to affect cell death (206). Recently, Yang et al. demonstrated that SHP-2 phosphorylation induced upon neutrophil activation by FcγRIIIb cross-linking results in reorganization within lipid rafts and causes a delay in activation-induced cell death (206). Thus, lipid raft association is crucial for receptor function since enhancement of FcγRIIa signaling is abrogated upon replacement of the FcγRIIIb lipid anchor with a transmembrane domain (207). On the other hand, it has been shown that cholesterol depleting reagents attenuate FcγRIIIb function resulting in upregulated activation of intracellular signaling molecules (203, 204).

## Modulation of Type II FC Receptor Function by the Lipid Cell Membrane

In comparison to type I FcRs, information about the role of membrane domains on type II FcR localization and function is scarce.

### Dectin-1

When it comes to pathogen recognition, signaling and the production of anti-microbial mediators such as reactive oxygen species via pattern recognition receptors, a phagocytic cell has to distinguish between soluble antigens released by pathogens in the distance and direct contact. This is particularly important when the target pattern recognition receptor can function as a monomer as is the case for Dectin-1, which does not require oligomerization for its activity *in vitro* [reviewed in (150)]. Hence, the question arises how Dectin-1 can distinguish between single microbial products and direct pathogen contact. This issue was resolved by Goodridge et al. providing evidence for the formation of a phagocytic membrane area. In contrast to soluble Dectin-1 ligands, particulate Dectin-1 target structures are able to induce Dectin-1 clusters and initiate signaling (208). Dectin-1 cluster formation in defined membrane areas can therefore be considered paramount to initiate direct anti-microbial programs only when needed. Xu et al. provided additional evidence supporting an impact of lipid membrane domains on Dectin-1. Sorting of Dectin-1 into lipid raft domains following stimulation with β-glucans coincides with the recruitment of syk and PLCγ2, while disruption of lipid rafts impairs the uptake of zymosan particles, signaling and cytokine production of murine bone-marrow derived DCs (209). As Dectin-1 was found to associate with FcγRIIb upon IgG immune complex binding (146) and FcγRIIb is localized to lipid rafts upon ligand-induced cross-linking (195) this further points toward an important role of organized microdomains for Dectin-1 activity.

### DC-SIGN

The priming of naïve T cell responses in secondary lymphoid organs requires the establishment of contact between DCs and T cells (210), a process which involves DC-SIGN (136). Once contact is established, communication takes place in a nanoscale area termed the immunological synapse (211). This microdomain concentrates molecules and ligands needed for a productive interaction and shaping of T cell immunity (211, 212). Following TCR and CD28 ligation, rafts are recruited to the contact area thereby enriching molecules responsible for signaling and contact maintenance (213). While the MHC:TCR interaction takes place in the inner zone of the formed synapse, outer zone adhesion molecules such as integrins maintain DC:T cell contact and sorting of these two molecule classes may be achieved by size exclusion of integrins to the inner zone (211, 213).

Initial studies on DC-SIGN neither assessed its localization within the cell membrane, nor the contribution of membrane domains for receptor function. In 2004, Cambi et al. first provided evidence for organization of DC-SIGN in membrane nanodomains likely to represent lipid rafts. Furthermore, these DC-SIGN assemblies were found to be important for binding and uptake of virus particles in moDCs (214). In a different study, DC-SIGN was found to form nanoscale clusters in living cells. These clusters localize to defined membrane areas and show little exchange with the surrounding membrane and are therefore assumed to be of stable nature (215). Furthermore, active movement of DC-SIGN clusters is observed with concentration at endocytosis sites (215). Therefore, the authors hypothesized that DC-SIGN is indeed organized in microdomain clusters, which move from the pathogen recognition site to the location of endocytosis (215). Finally, lipid raft localization of DC-SIGN was confirmed by co-precipitation with downstream signaling molecules upon receptor cross-linking (216).

### FcεRII

The assembly of B cell receptor containing lipid rafts is a crucial component of B cell development and activity (90, 91, 217). Even though data on the localization of FcεRII within defined membrane domains are so far lacking, the importance of FcεRII expressed on B cells was recently demonstrated. During the process of affinity maturation, ligation of FcεRII by IgG induces the upregulation of FcγRIIb thereby raising the threshold for BCR signaling (173). However, if this involves coalescence of FcεRII, the BCR and FcγRIIb within lipid rafts has to be determined.

## Clinical Implications of Plasma Membrane Manipulation for FCR Function

Based on the crucial role of the plasma membrane and its organized domains for many cellular processes, drugs altering the plasma membrane lipid composition, structure or function have been developed for, e.g., malignant, metabolic, cardiovascular, or neurodegenerative disorders (218, 219). One example for such a drug, the compound azurin, has recently been shown to decrease the plasma membrane order of cancer cells upon uptake thereby attenuating cell signaling and subsequently tumor cell growth (220). In addition, plasma membrane altering functions have been described for established therapeutics such as lovastatin, a cholesterol-reducing drug applied in the treatment of hypercholesterinemia (221, 222), or non-steroidal anti-inflammatory drugs (NSAIDs) used to treat inflammatory disorders which were shown to cause membrane softening and re-organization of membrane associated proteins (223, 224). Assuming that FcγR function is indeed regulated by the plasma membrane lipid environment, it is very likely that these drugs may alter FcγR and thus IgG activity. Of note, this may not only affect immune cells but also non-immune cells which can also express FcRs [summarized in (225)]. For instance, human endothelial cells express both DC-SIGN (226, 227) and FcγRIIb (228, 229) which is involved in trafficking of maternal IgG (230) or clearance of small IgG immune complexes in liver sinusoids (231). Furthermore, FcγRs have been identified on cells of the human central nervous system including neurons and microglia, i.e., brain specific phagocytic cells. Microglia express all types of FcγR (232) which are also found to be upregulated under inflammatory conditions (233). Neurons express FcγRIa and FcγRIIb that are involved in IgG uptake and induce the release of neurotransmitters (234). Taken together, it is reasonable to speculate that a treatment with drugs affecting the plasma membrane might also affect FcR dependent effector functions of IgG on immune and non-immune cells *in vivo*. More research identifying these potential systemic drug side-effects will be required to understand this in more detail in the future.

## Concluding Remarks

Lipid cell membranes are highly complex mixtures of different lipids and proteins that can interact with and influence each other. Moreover, lateral heterogeneity of membranes including the formation of organized microdomains, i.e., lipid rafts, have previously been shown to affect a number of receptors expressed on immune cells. It is therefore reasonable to assume that distinct membrane localization is also involved in the modulation of FcR function. However, the respective experimental data is both, incomplete (e.g., with respect to FcR expression on different cell types or impact of membrane domains on FcR-IgG interactions and subsequent effector functions) and partially inconsistent. One possible explanation for the observed inconsistencies might be the use of various cell lines with possibly divergent membrane compositions in relation to primary cells. Furthermore, different experimental approaches might also affect the results and interpretation of the data. For example, cold non-ionic detergent extraction was once widely used to investigate the membrane structure of organized domains and their function [reviewed in (26, 235, 236)]. It was later, however, demonstrated that detergents diminish the lateral complexity of membranes. Therefore, detergent-resistant membrane structures seem to be artificial and not reflecting the native structure of lipid rafts in living cells [reviewed in (237)]. In recent years, membrane manipulation has gained popularity in analysis of complex membrane compositions and the significance of organized domains. The most commonly used method to manipulate cellular membranes is based on cholesterol depletion by chemical reagents. Nevertheless, targeting cholesterol with various chemicals to disrupt lipid raft organization seems to differentially affect the cell membrane necessitating cautiousness when interpreting experimental data. Furthermore, the biological effects of these chemicals have not been investigated in great detail [reviewed in (40, 180)]. In addition, present technical advances in the field of high-resolution microscopy allow analyzing the impact of native cell membranes, especially lipid rafts, on the localization of membrane receptors such as FcRs in relevant cellular contexts. One, however, must be aware that fixation of samples for microscopy might alter receptor location and plasma membrane structure (238, 239). In conclusion, future research will need to clarify the role of lipid cell membranes and organized membrane domains for type I and type II FcR function and hopefully allow implementing some of these findings for developing novel therapeutic avenues to enhance or suppress FcR dependent effector functions.

## Author Contributions

AL and DD drafted the manuscript. SK, LA, JL, and AL wrote the manuscript. FN and DD revised the manuscript. All authors critically reviewed the manuscript.

## Conflict of Interest

The authors declare that the research was conducted in the absence of any commercial or financial relationships that could be construed as a potential conflict of interest.
